# Statistical Challenges in Risk Prediction

**DOI:** 10.1002/bimj.201500045

**Published:** 2015-06-05

**Authors:** Ian R. White, Angela M. Wood

**Affiliations:** ^1^ Medical Research Council Biostatistics UnitCambridge Institute of Public HealthCambridgeCB2 0SRUK; ^2^ Cardiovascular Epidemiology UnitDepartment of Public Health and Primary Care, University of CambridgeCambridgeCB1 8RNUK

##  

**Figure 1:**
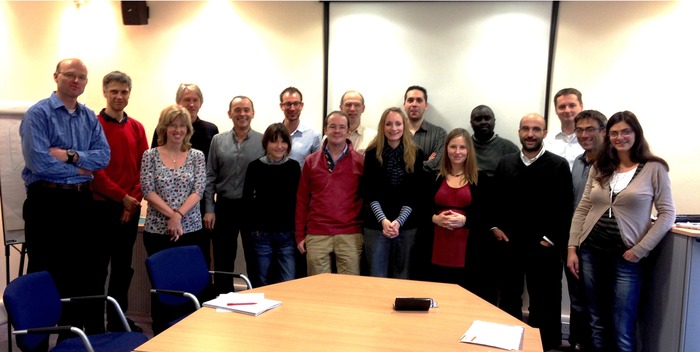
Seventeen of the 21 invited delegates of the international workshop on Statistical Challenges in Risk Prediction in Cambridge, November 2012 are shown.

Risk prediction is an important area of clinical application of statistical methods. Angela Wood, assisted by Ian White and Simon Thompson, organized a three‐day international workshop on Statistical Challenges in Risk Prediction in Cambridge, UK, on November 19–21, 2012, with 21 invited academic statisticians (Fig. 1). The workshop covered statistical challenges faced when developing, assessing, and validating risk models for generic clinical use and in particular for cardiovascular risk prediction. The papers in this special issue (which went through the usual peer review process) represent some of the presentations and discussions from the four workshop sessions.

The first workshop session was on development of risk prediction models and included presentations and discussion on model stability issues, using repeated measures of risk factors, and how to choose between age and study duration as the timescale. The session is represented here by the paper of Sauerbrei et al., “On stability issues in deriving multivariable regression models”. A selected model is “stable” if it is unchanged by moderate changes to the data. An unstable model may yield stable predictions, but it may be sensitive to influential data points. Stability is assessed by re‐applying the model selection procedure to multiple bootstrap samples drawn from the observed data. Graphical displays are proposed to explore the issue and highlight possible influential data points.

The second workshop session was on assessment of risk prediction models. This is represented here by three papers based on presentations and discussion from the workshop. “Graphical assessment of incremental value of novel markers in prediction models: From statistical to decision analytical perspectives” by Steyerberg et al. is a timely and important review of available methods, from graphs of predictions through measures of explained variation to methods based on reclassification. In addition, it proposes the net reclassification risk graph as a new graphical presentation. The other two papers are more technical and focus on new measures of explained variation and discrimination. “Explained variation for recurrent event data” by Alotaibi et al. extends measures of explained variation used for standard survival data to the setting of recurrent events, illustrated by data on episodes of infant diarrhea. “Covariate‐adjusted measures of discrimination for survival data” by White and Rapsomaniki argues that covariate adjustment is needed to make discrimination measures comparable across studies, and proposes appropriate methods.

The third workshop session was on current challenges in cardiovascular risk prediction and focused on individual participant data meta‐analysis, risk prediction using case‐cohort and nested case–control studies, multiple imputation and prediction, model validation, and optimizing sequential screening. The session is represented here by “The estimation and use of predictions for the assessment of model performance using large samples with multiply imputed data” by Wood et al., which explores the difficulties of risk prediction in a cardiovascular or other setting when predictors are incomplete. “Ideal” and “pragmatic” model performances are distinguished, and methods are proposed to estimate both.

The final workshop session was on evaluating the clinical value of risk models and included presentations on measures to quantify public health impact, including decision curve analysis, and on the economics of risk prediction. No paper from this session appears in this special issue, although Steyerberg et al. (described above) discuss decision curve analysis. Discussion during the workshop concluded that more methods development is needed to advance the evaluation of risk models in the clinical context in which the models will be used, and hence to evaluate the patient benefit and economic benefit of using the risk models in clinical practice.

We hope you will enjoy reading these papers and contributing to the development of this important area of biostatistical methodology. We thank the *Biometrical Journal* and its editors and reviewers for allowing us to publish the papers together in the journal.

Angela Wood, Ian White

Guest Editors

